# Atherogenesis, Transcytosis, and the Transmural Cholesterol Flux: A Critical Review

**DOI:** 10.1155/2022/2253478

**Published:** 2022-04-14

**Authors:** Doron Goldberg, Soliman Khatib

**Affiliations:** Laboratory of Natural Compounds and Analytical Chemistry, MIGAL - Galilee Research Institute, Kiryat Shmona 11016, And Tel-Hai College, Upper Galilee 1220800, Israel

## Abstract

The recently described phenomenon of cholesterol-loaded low-density lipoproteins (LDL) entering the arterial wall from the lumen by transcytosis has been accepted as an alternative for the long-held concept that atherogenesis involves only passive LDL movement across an injured or dysfunctional endothelial barrier. This active transport of LDL can now adequately explain why plaques (atheromas) appear under an intact, uninjured endothelium. However, the LDL transcytosis hypothesis is still questionable, mainly because the process serves no clear physiological purpose. Moreover, central components of the putative LDL transcytosis apparatus are shared by the counter process of cholesterol efflux and reverse cholesterol transport (RCT) and therefore can essentially create an energy-wasting futile cycle and paradoxically be pro- and antiatherogenic simultaneously. Hence, by critically reviewing the literature, we wish to put forward an alternative interpretation that, in our opinion, better fits the experimental evidence. We assert that most of the accumulating cholesterol (mainly as LDL) reaches the intima not from the lumen by transcytosis, but from the artery's inner layers: the adventitia and media. We have named this directional cholesterol transport transmural cholesterol flux (TCF). We suggest that excess cholesterol, diffusing from the avascular (i.e., devoid of blood and lymph vessels) media's smooth muscle cells, is cleared by the endothelium through its apical membrane. A plaque is formed when this cholesterol clearance rate lags behind its rate of arrival by TCF.

## 1. Introduction

According to the accepted paradigm, atherogenesis is initiated by a lesion in the large arteries' endothelium, caused mainly by oxidative stress and inflammation. This results in endothelial dysfunction and increased permeability [1]. Cholesterol-carrying lipoproteins in the blood, mostly LDL, then enter from the lumen by passive filtration through the breached endothelial monolayer and accumulate in the subendothelial intima [2]. However, developing plaques appear under intact endothelium, with no visible injury [3], and passive LDL filtration through paracellular or transcellular pores or channels in an injured endothelium has never been conclusively demonstrated [4]. Therefore, the exact mechanism of LDL entry into the intima has remained an unsolved puzzle for decades.

Now, it seems that this puzzle has finally been solved by several research groups, who have recently provided experimental evidence for the active transport of LDL, as well as high-density lipoproteins (HDL), by transcytosis from the apical endothelial membrane to the basolateral membrane [4–6]. This transcytosis mechanism operates in intact, healthy endothelial cells, thereby rendering the invocation of passive filtration and endothelial injury to explain atherogenesis initiation unnecessary.

Most of the experimentally demonstrated endothelial transcytosis of lipoproteins is specific and mediated by at least two cell-surface receptors - scavenger receptor class B, type 1 (SR-B1) and activin receptor-like kinase 1 (ALK1) [7–11]. The only exception is the blood-brain barrier, where LDL transcytosis is mediated by the LDL receptor (LDLR) [12]. SR-B1 is a dimer, identified primarily as an HDL receptor and a bidirectional cholesterol transporter of the liver and peripheral tissues [13, 14]. It binds LDL and HDL with similarly high affinity; however, HDL binding blocks LDL binding, but not vice versa [15]. ALK1 is an endothelial-specific TGF-*β* type 1 receptor involved in angiogenesis [11, 16], whereas SR-B1 transports both LDL and HDL, ALK1 transports only LDL [11].

Both SR-B1 and ALK1 are localized to the caveolae - cholesterol-rich, flask-shaped invaginations of the cell membrane. Knockout of the genes for either SR-B1 (*Scarb1*) or ALK1 (*Alk1*), or knockdown of their expression, can attenuate endothelial LDL and HDL transcytosis in vitro by 30-50% [8–11]. Endothelial-specific knockout of *Scarb1* significantly reduces the extent of atherosclerotic lesions in the aorta of apolipoprotein E-deficient (*apoE^−/−^*) mice [9]. Similarly, knockout of the gene for caveolin-1 (*Cav1*), a major component of the caveolae, eliminates the caveolae, decreases LDL transcytosis in vitro by 50%, and dramatically reduces the area of atherosclerotic lesions in *apoE^−/−^* or LDLR-deficient (*Ldlr^−/−^*) mice [17–19]. Conversely, the endothelial-specific overexpression of *Cav1* in *apoE^−/−^* mice accelerates atherosclerosis [20].

Cholesterol is an essential component of the plasma membrane, maintaining its integrity and fluidity and regulating oxygen diffusion through it [21, 22]. Hence, cholesterol homeostasis must be tightly controlled. However, cholesterol is non-nutritive, and surplus cholesterol, unlike most other metabolites, cannot be discarded by oxidation to CO_2_ and water [21]. Therefore, to prevent toxic cholesterol accumulation, surplus cholesterol must be removed. This clearance is achieved by a process termed reverse cholesterol transport (RCT), whereby excess cholesterol is actively collected by HDL and transported, at the expense of energy, first by the lymphatic system and then by the bloodstream to the liver and small intestine for recycling, bile production, or final elimination in the feces [23].

Endothelial transcytosis of HDL can be readily construed as an RCT mechanism for the increased clearance of cholesterol from the intima. HDL transported by transcytosis can efflux excess cholesterol from intimal macrophages. In diseased atherosclerotic arteries, where adventitial lymphatic vessels expand and grow in number, the cholesterol-loaded HDL particles can then find their way back to the circulation through arterial lymphatics [24, 25]. Yet, intimal HDL particles in normal arteries probably return to the circulation by diffusion and transcytosis in the reverse basolateral-to-apical direction [7, 26]. Vaisman et al. [7] speculated that cholesterol from intimal HDL particles can be taken up by basolateral SR-B1 receptors and then transported across the endothelial cells as free cholesterol, to be picked up again by apical SR-B1 receptors and plasma HDL.

Unlike HDL transcytosis, the experimentally observed apical-to-basolateral LDL transcytosis serves no known physiological purpose. Since the rate of cholesterol influx into the arterial wall is much faster than its retention rate during atherogenesis (by three orders of magnitude according to one estimate [27]), its removal must be equally fast to prevent accumulation [27, 28]. The corollary is that if LDL transcytosis is active under normal conditions and is responsible for this rapid influx, the arterial endothelium operates a rapid and delicately balanced futile cycle that wastes energy only to recycle large amounts of cholesterol through the intima.

Moreover, in the context of LDL transcytosis, endothelial-specific manipulations of the SR-B1 expression create results that are contradictory and hard to interpret: whereas the endothelial-specific knockout of *Scarb1* in *apoE^−/−^* mice reduces atherosclerosis [9] - ostensibly due to reduced LDL transcytosis-its endothelial - specific overexpression, on a similar *apoE*^−/−^ background, is also antiatherogenic [7]. On the other hand, complete (whole-body) knockout of *Scarb1* increases atherosclerosis in *apoE*^−/−^ mice (*Scarb1*^−/−^, *apoE*^−/−^) as a result of impaired RCT [29], and humans who are homozygous for a rare loss-of-function mutation of *Scarb1* are 1.8 times more likely to develop coronary heart disease [30]. Hence, the SR-B1 receptor, an essential component of the RCT apparatus, can be simultaneously anti- and proatherogenic - a paradox that is admittedly difficult to reconcile [6, 9, 31].

It seems that the hypothesis of LDL transcytosis solves one puzzle while creating a new one. Our way out of this unclear state of affairs is through the arterial wall's backdoor of the adventitial vasculature - the vasa vasorum (vv) [32]. By extensively reviewing the existing evidence, we could reasonably conclude that most lipoproteins enter the wall of large arteries not from the lumen by transcytosis but through the vv and diffuse through the media and intima till they reach the basolateral endothelial membrane. Excess cholesterol in the intima is then cleared and returned to the circulation by the endothelium through its apical membrane. Atherogenesis is, therefore, a consequence of an imbalance in the transmural cholesterol flux (TCF) between the rate of cholesterol influx through the vv and its rate of clearance by the endothelium. This alternative interpretation of the experimental evidence readily incorporates lipoprotein transcytosis and also takes into account the unique isolation of the vascular smooth muscle cells (VSMCs) in the avascular media layer [33], the involvement of medial VSMCs in the early stages of plaque formation and generation of foam cells [34], the involvement of SR-B1 in angiogenesis [35, 36], and the dependence of the murine *apoE^−/−^* model of atherosclerosis on vv neovascularization [37, 38].

## 2. Reviewing the Evidence

### 2.1. The Nexus of Avascular Media, Vasa Vasorum, and Atherosclerosis

The media layer of VSMCs is mostly avascular and partially isolated by elastic laminas. This isolation can render the multilamellar media of large arteries “immune-privileged” and challenging to treat when infected [33, 39]. Therefore, the media can rely only on the diffusion of oxygen and nutrients - either from the vessel's lumen or from the perivascular space - through the respective adjacent layers of the intima and adventitia [40]. This diffusion is sufficient for small-diameter blood vessels, with a wall thinner than 29 cell layers [32]. In thick-walled vessels, however, diffusion from the outer layers cannot sustain the inner avascular media, and it must be assisted by adventitial vv to supply oxygen and nourishment by diffusion to medial VSMCs [40]. In large animals' aortas, where diffusion from the vv is insufficient, the vv expands from the adventitia into the proximal media layers. However, media layers closer to the intima remain avascular and dependent on transendothelial diffusion [40]. In general, vessels with a lumen diameter of less than 0.5 mm, such as all normal vessels in mice and intramyocardial vessels in humans, do not have vv [32].

The vv and atherosclerosis are intimately linked [41]. In humans and model animals, vv vessels' density correlates with atherosclerosis progression and the extent of atherosclerotic lesions [42, 43]. Intramyocardial arteries that lack vv are atheroresistant, even under the most proatherogenic conditions [44, 45]. Extensive neovascularization and extension of the vv deeper into the media and intima usually precede plaque formation [46]. In atherosclerotic *apoE*^−/−^ and *ldlr*^−/−^ mice, inhibiting vv neovascularization by antiangiogenic agents reduces atherosclerosis and the extent of atherosclerotic lesions [37, 38]. Since the murine aorta does not normally have vv, the implication is that these mouse models are dependent on angiogenesis and vv neovascularization. In this regard, the increased propensity of *apoE*^−/−^ mice to develop atherosclerosis (compared with *ldlr*^−/−^ mice) and the potent inhibition of endothelial proliferation by human ApoE are probably not coincidental [47, 48].

Another link between atherosclerosis and the vv becomes evident when physical occlusion of the vv induces ischemia and necrosis of the media [49], as well as rapid focal accumulation of cholesteryl ester (CE) in the underlying intima [50] - probably as a result of increased hypoxia-induced cholesterol esterification [51] and extensive VSMCs death and disintegration. This intimal accumulation implies that the arterial exit gate for excess cholesterol is the intima rather than the adventitia with its limited lymphatic system [52].

It seems that the vv is a mixed blessing for large, thick-walled arteries. While it helps supply oxygen and nutrients to the inner avascular media, it can also increase the influx of surplus cholesterol into the arterial wall, which cannot be easily discarded due to the lack of lymph vessels in the media and their limited number in the adventitia under normal conditions [52]. This precarious situation can make large arteries more vulnerable to cholesterol homeostasis disorders than other peripheral tissues [53, 54]. For example, humans who are homozygous carriers of an LDLR null mutation that causes familial hypercholesterolemia have extremely high blood cholesterol and suffer from early onset of atherosclerosis and tendon xanthomas [21]. Nonetheless, the rest of their organs and tissues remain essentially unharmed.

### 2.2. The Transmural Cholesterol Flux Runs from the Adventitia to the Endothelium

**Figure 1:**
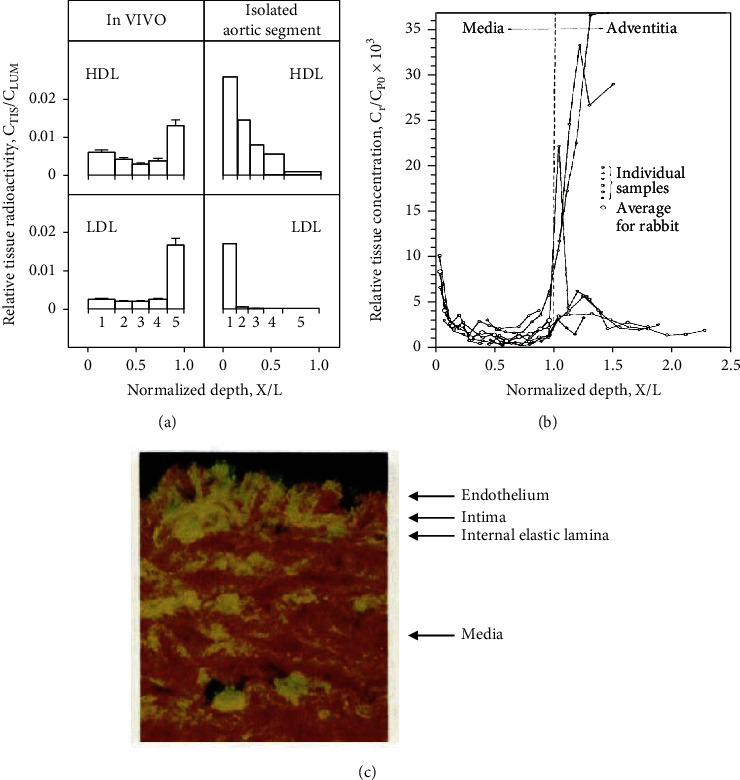
The entry of lipoproteins into the aorta wall. In all the examples below, the original authors concluded that lipoproteins enter the wall mainly from the lumen, but our alternative interpretation also fits the data. (a) The relative ([tissue]/[plasma]) concentration profile of radiolabeled cholesteryl ester in a pig aorta wall after administrating a pulse of differentially radiolabeled HDL and LDL either in vivo (left panel, 6.5 hours tissue exposure) or in situ into an isolated aortic segment (right panel, 4 hours tissue exposure). *X* is the lumen-tissue slice distance, and *L* is the wall thickness. The numbers 1-5 represent the aortic layers from the luminal to the adventitial side. The U-shaped concentration profile, spanning the entire wall thickness, is formed only in the in vivo experiment when lipoproteins can also enter the aorta wall through the vv. The minimal ability of LDL to penetrate the wall beyond the intima when the labeled lipoproteins cannot enter through the vv (right panel, bottom) is also evident. The observed “damming” of LDL at the intima-media boundary is attributed to the internal elastic lamina [57] but may represent the binding and trapping of LDL by intimal extracellular matrix proteins (adapted from Nordestgaard et al. [57]). (b) The relative ([tissue]/[plasma]) concentration profile of ^125^I-labeled ApoB in the rabbit aorta wall 10 min. After an i.v. administration of iodinated LDL, *X* is the lumen to mid-tissue slice distance, and *L* is the lumen to medial-adventitial boundary distance. Each line represents a separate experiment. The transmural U-shaped concentration profile of ApoB, or its degradation products, is evident (adapted from Bratzler et al. [55]). (c) The fluorescence of FITC-labeled anti-LDL antibody in a section of rhesus monkey aorta wall 6 hours after administering 1gr/kg cholesterol by a gastric tube. The presence of the lipoprotein in the subendothelial and medial layers can be clearly seen by the greenish-yellow coloring of the tissues (adapted from Shimamoto et al. [60]).

In a series of in vivo experiments during the 1970s and 80s in human subjects, pigs, and rabbits, radiolabeled cholesterol and lipoproteins were injected intravenously to gain insight into the mechanism by which plasma cholesterol enters the arterial tissues [28, 55–58]. The resulting concentration profile of the radiolabeled LDL apolipoproteins and cholesterol in the aorta wall was found to be unevenly U-shaped, with the lowest concentration in the media and the highest concentration in the adventitia, rather than the intima (Figures 1(a) and 1(b)). This concentration profile and the penetration of LDL proteins deep into the media and adventitia layers were also demonstrated using specific anti-apolipoprotein B (ApoB) antibodies (Figure 1(c)) [59–61]. In rabbits, the U-shaped concentration profile of ^125^I-LDL was rapidly established (within 10 min) in the aorta (Figure 1(b)) [55]. Similarly, the rate of influx of CE into healthy human aortic tissue was so high compared to the tissue's cholesterol content that its rapid removal was found to be essential for preventing cholesterol deposition [56].

Using the “pulse-chase” technique with radiolabeled LDL and HDL, Nordestgaard, Hjelms, and colleagues [57, 58] measured cholesterol entry into the aorta wall of living pigs. They also observed the uneven U-shaped concentration profile of labeled cholesterol, with the highest concentration in the adventitia (Figure 1(b)) [57, 58]. During the in vivo cold “chase,” the concentration of labeled HDL CE, which was found throughout the wall, decayed more rapidly in the intima (our interpretation of [57, 58]). When the experiment was repeated in situ, with labeled lipoproteins injected into the lumen of an isolated aortic segment (Figure 1(b)), very little LDL CE could penetrate the wall beyond the intima, whereas the HDL CE reached the adventitia [57].

The interpretation of this phenomenon by Nordestgaard et al. [57] was that CE in LDL and HDL enters the aortic wall from both the luminal and adventitial sides but leaves through the adventitial vv and lymphatics. Nonetheless, an equally plausible interpretation of these results is that under normal in vivo conditions, plasma lipoproteins rapidly enter the aorta wall mainly through the adventitial vv and exit back to the bloodstream through the endothelium. The U-shaped profile indicates that most lipoproteins entering the media quickly leave and are not retained. This latter interpretation implies unidirectional adventitia-to-intima TCF. It takes into account the high rate of cholesterol influx into the arterial wall, the limited ability of LDL CE to cross the intima from the lumen [57], the transmural U-shaped concentration profile of labeled cholesterol, and the scarcity of lymph vessels in the normal adventitia [52]. The mechanism accounting for the rapid cholesterol influx could be passive ultrafiltration of lipoproteins through caveolae-based transendothelial channels in the vv capillaries' endothelium [62, 63], which might be structurally very different from the endothelium of the large arteries [64].

Although the highest concentration of intravenously injected labeled lipoproteins was found in the adventitia, the resulting U-shaped concentration profile is incompatible with diffusion-driven TCF in any direction. However, the high intimal concentration of labeled lipoproteins probably included lipoproteins that were directly bound to the luminal endothelial surface or had been internalized by receptor-mediated endocytosis. It is also known that a significant fraction of the intimal cholesterol and lipoproteins is immobilized [65] and cannot freely diffuse due to interaction with extracellular matrix proteins such as collagen [66], elastin [67], fibrin [68], and proteoglycans [69]. This immobilization could be a mechanism to secure the adventitia-to-endothelium cholesterol concentration gradient and the continuous inward cholesterol flux from the media towards the endothelium, even when efflux from the apical endothelial membrane is disturbed.

### 2.3. Lipoproteins Transcytosis Can Be Bidirectional

While it is not always the case, the experimentally demonstrated HDL transcytosis could be bidirectional and run from the apical endothelial membrane to the basolateral membrane or in the reverse (basolateral-to-apical) direction [7]. Rohrer et al. [8] found only unidirectional apical-to basolateral HDL transcytosis. On the other hand, Vaisman et al. [7] found that more SR-B1 is expressed on the basolateral membrane and that basolateral-to-apical HDL transcytosis also occurs, albeit at a slower rate. The normal RCT from peripheral tissues through the lymphatic system, which probably handles most of the body's returning cholesterol, also involves basolateral-to-apical HDL transcytosis through the lymph vessels' endothelium [70].

Unlike HDL transcytosis, LDL transcytosis has been demonstrated only in the apical-to-basolateral direction [9–11, 71]. However, it is unclear whether LDL transcytosis cannot occur in the reverse direction or whether this control experiment has not been tried. Thus, on the face of it, there is no a priori reason to assume that LDL transcytosis, unlike its HDL counterpart, is not bidirectional, and cannot transport LDL from the basolateral endothelial membrane to the apical membrane as well. A simple transwell assay could, and should, settle this question.

### 2.4. Endothelium-Specific Mutations of Cholesterol Transporters Affect Atherogenesis

The cholesterol transporters ATP-binding cassette transporter A1 (ABCA1) and G1 (ABCG1) are essential components of cholesterol efflux and RCT [72]. Endothelium-specific knockout of their genes accelerates diet-induced atherogenesis in *ldlr*^−/−^ mice [73], whereas the endothelial overexpression of human ABCA1 in *apoE^−/−^* mice slows it down [74]. Induction of the endothelial expression of both ABCA1 and ABCG1 by an agonist of the nuclear receptor liver X receptor (LXR) also correlates with reduced atherosclerosis in mice [75]. Similarly, the exclusively endothelial overexpression of the HDL receptor and cholesterol transporter SR-B1 in *apoE^−/−^* mice can also reduce diet-induced atherosclerosis [7].

### 2.5. Most Foam Cells in the Plaque Originate from VSMCs

The implication of medial VSMCs and their cholesterol homeostasis in atherogenesis comes from the recent observation that most foam cells in the plaque (>70% in mice and >50% in humans) originate not from infiltrating monocytes but from transdifferentiating VSMCs [34, 76, 77]. In humans, this VSMC transformation seems to start already in utero, when a thick layer of VSMCs develops in the intima of atherosclerosis-prone arteries [76, 78], probably due to chronic exposure to excess cholesterol [79]. The thickened intima, termed diffuse intimal thickening (DIT), also contains elastin and proteoglycans and is initially devoid of cholesterol deposits, which appear only at a later stage [69].

### 2.6. Plaques Start Deep at the Media-Intima Boundary and Progress Inward under an Intact Endothelium

Plaques develop in DITs, mainly in places where blood flow is turbulent [80]. The earliest deposits of LDL cholesterol and ApoB appear with no sign of inflammation or the presence of macrophages [69]. They can be initially detected mainly in the deep layer of the media-intima boundary [66, 69], instead of close to the basolateral endothelial membrane, as would be expected if this LDL arrived from the lumen by endothelial transcytosis. The accumulating lipids progress inward towards the endothelium and eventually fill the intima and appear as “fatty streaks” under an intact endothelium, which remains so throughout most stages of plaque progression [3]. This preferential ApoB accumulation near the media-intima boundary instead of immediately below the endothelium has also been observed in well-developed plaques [59, 61, 81].

## 3. Tying It All Together

### 3.1. A Detailed Account of TCF-Driven Atherogenesis

**Figure 2:**
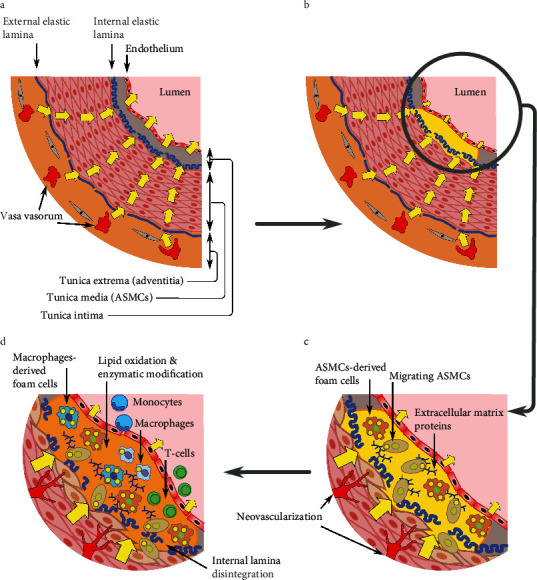
Transmural cholesterol flux-driven plaque formation. (a) Cross-section through a healthy artery with fully functioning reverse cholesterol transport (RCT). The yellow arrows represent transmural cholesterol flux (TCF) of lipoproteins and cholesterol from the vasa vasorum (vv) in the adventitia, maybe through fenestra in the elastic laminas, to the vascular smooth muscle cells (VSMCs) in the avascular media, and finally to the apical endothelial membrane. (b) The result of impaired endothelial RCT: as the intima strives to maintain the TCF, excess cholesterol slowly accumulates in the subendothelial layer by binding to intimal extracellular matrix proteins [131] and initiates plaque formation. (c) A magnified cutaway of the initial plaque shows smooth muscle cells migrating from the media into the intima and their transdifferentiation into cholesterol-loaded foam cells [76]. The hypoxia in the underlying media, caused by the increased endothelial cholesterol concentration, stimulates neovascularization and vv expansion [41]. Both VSMCs migration into the intima and vv expansion can precede plaque formation [76, 118]. (d) The oxidation and enzymatic modification (i.e., production of enzymatically modified LDL‒eLDL) of the accumulating lipids in the growing plaque trigger the immune system [84, 85] and the consequent inflammation and migration of monocytes and T-cells from the lumen. The monocytes differentiate into cholesterol scavenging macrophages which eventually become foam cells [120]. This inflammation marks the formation of a full-fledged atherosclerotic plaque.

The evidence upon which our assumption of TCF-driven atherogenesis is based can be summarized as follows:

- The media layer of VSMCs is avascular, partially isolated, and must rely only on diffusion for oxygen and nutrients supply. Consequently, lipoproteins and cholesterol can only enter and leave the media by diffusion
- Cholesterol, carried by lipoproteins, enters the wall of large arteries mainly through the capillaries of the adventitial vv and reaches the media by diffusion (Figure 2(a))
- Excess cholesterol in the avascular media can only leave by diffusion down its concentration gradient towards the intima. It crosses the endothelium assisted by receptor-mediated endocytosis and basolateral-to-apical transcytosis of LDL and HDL. It is then cleared through the endothelium's apical membranes by several efflux mechanisms, including the HDL-mediated efflux that is specifically adapted to this unique environment
- Lipoproteins entering the intima from the lumen, by either active transport or pressure-driven convection through temporary breaches in the endothelium (i.e., dying or dividing cells), are also cleared and returned to the bloodstream by the endothelium
- The transmural cholesterol concentration gradient points from the adventitia to the intima. It is maintained by the constant influx of cholesterol through the vv and its synthesis in the media on the one hand and the continuous efflux of cholesterol back to the bloodstream through the apical endothelial membrane on the other. The intima's capacity to sequester cholesterol also helps maintain this transmural gradient
- The endothelial surface area-to-wall thickness ratio determines the endothelial membrane's capacity to sustain the inward cholesterol flux from the VSMCs. In large arteries, with a thick media layer and rapid cholesterol influx through the vv, this capacity becomes relatively smaller
- The reliance of medial VSMCs on cholesterol diffusion and removal through the “bottleneck” of the apical endothelial surface makes the arteries exceptionally vulnerable to cholesterol homeostasis disorders
- Atherogenesis is initiated when the cholesterol flux from the VSMCs becomes faster than its clearance through the apical endothelial membrane (Figure 2(b)). Since the accumulating cholesterol is effectively sequestered and immobilized by intimal extracellular matrix proteins, the concentration gradient of freely diffusing cholesterol and lipoproteins is maintained, and the TCF can continue
- Factors that tip the balance in favor of endothelium-bound TCF (such as increased blood cholesterol and vv neovascularization and expansion) are likely to be proatherogenic; factors that tip it toward endothelial efflux (such as higher levels of efflux-efficient HDL and reduced blood pressure) are likely to be antiatherogenic

### 3.2. The Endothelial HDL-Mediated Cholesterol Efflux and Plaque Formation

The Achilles' heel of HDL-mediated RCT through the apical endothelial membrane is the binding of HDL to its SR-B1 receptor while facing the force of the flowing blood. In places where the flow is laminar, this force is minimal. However, near arterial curvatures or branching, the flow becomes turbulent, and the bound HDL particle may have to resist its full force, which increases rapidly as the particle's diameter grows. To keep the HDL bound, the endothelial RCT apparatus deploys two essential and effective countermeasures: (1) lowering blood pressure through eNOS activation by SR-B1 upon HDL binding [13] and (2) a gradual increase in HDL's binding affinity to SR-B1 as the particle grows [72].

The activation of eNOS by HDL binding to SR-B1 and by the HDL-associated sphingosine 1-phosphate molecules binding to their receptor (S1PR_3_) serves to maintain endothelial homeostasis and integrity [82]. Nevertheless, it seems that this activation, together with the gradually increasing HDL binding affinity, also represents a specific adaptation of the HDL-mediated RCT to the environment of the apical endothelial membrane of blood vessels.

A disruption in the balance between this HDL-mediated cholesterol efflux and its influx into the arterial wall will result, over time, in cholesterol accumulation in the intima - mainly as LDL arriving through the vv (Figure 2(b)). The intima's known ability to sequester cholesterol, which could maintain its concentration gradient, now facilitates its accumulation [66, 69, 83]. Once plaque formation begins, it will proceed (Figures 2(c) and 2(d)) through the well-documented stages of atherogenesis, such as proinflammatory lipid oxidation [84] and LDL enzymatic modifications (eLDL generation) [85], inflammation, and immune cells' infiltration [1].

Atherosclerosis is a slowly progressing disease that takes many years to develop, even in patients with severe cholesterol homeostasis disorders such as Tangier disease (ABCA1 loss of function), who have no or very little HDL [86]. Surprisingly, the amount of excreted sterols in the feces of Tangier disease patients or ABCA1-deficient mice remains normal, and they show no disturbance in whole-body cholesterol homeostasis [87]. Therefore, it seems that, like any other mission-critical system, the RCT apparatus has a high degree of redundancy and the ability to operate in parallel with or activate one or more backup systems [54]. For example, Hung et al. [88] showed that in *apoA1*-knockout mice, with no HDL-mediated RCT, injected cholesterol-loaded foam cells transferred most of their labeled cholesterol to red blood cells, which then carried most of it to the liver. However, due to their unique physiology (as explained above), disturbances in HDL-mediated RCT in the large arteries inevitably result in the slow formation of plaques, despite the apparent existence of alternative efflux mechanisms in the endothelium, the identity of which is still largely unknown.

### 3.3. How Does Endothelial Transcytosis of Lipoproteins Fit in?

According to our TCF assumption, the endothelium is responsible for clearing surplus cholesterol arriving from the arterial wall and is well adapted to this task [89]. Therefore, the endothelial transcytosis mechanism is an essential component of arterial RCT. We assume that under normal physiological conditions, it transports lipoproteins mostly from the basolateral endothelial membrane to the apical membrane, like the transcytosis of HDL from peripheral tissues into the lymph [70]. The transcytosis of HDL in the reverse apical-to-basolateral direction can also be, as explained above, an integral part of the cholesterol-clearing process [7].

On the other hand, apical-to-basolateral LDL transcytosis does not easily fit this paradigm. Although it can be used to supply LDL cholesterol to medial VSMCs [4], this possibility seems unlikely since VSMCs (similar to other cells) can synthesize their own supply [90] or get it through the vv. It is also possible that the transcytosed LDL, like HDL, functions as a cholesterol sink in the subendothelial intima [91] and is then transcytosed in the opposite direction or reabsorbed by the endothelium through receptor-mediated uptake. In any case, if this intima-bound LDL transcytosis is physiologically significant, its actual function may be revealed in future research. However, we suggest that the preferred direction of LDL transcytosis is also basolateral-to-apical as part of the endothelial RCT. The transport in the opposite direction observed in vitro might be a consequence of nonphysiological experimental conditions, such as the complete absence of competing HDL [31] or an unnaturally steep LDL concentration gradient across the transwell assay system used in such experiments [71].

To date, the in vivo evidence for apical-to-basolateral LDL transcytosis is only circumstantial and therefore questionable, since the key experiments [9, 18] could not differentiate between LDL entering the intima by transcytosis from the lumen and LDL arriving through the vv route. We believe that the modest size reduction in atherosclerotic lesion area observed in mice with endothelial-specific knockout of *Scarb1* [9] resulted not from diminished LDL transcytosis but from reduced neovascularization. SR-B1 has proangiogenic activity [35, 36], and the *apoE^−/−^* murine model is dependent on angiogenesis and vv neovascularization [37, 38]. Therefore, the endothelial-specific *Scarb1* knockout may have reduced aortic neovascularization and consequently, reduced atherosclerosis.

This interpretation of the experimental evidence can solve the apparent paradox of SR-B1 being simultaneously anti- and proatherogenic [31]. The endothelial-specific *Scarb1* overexpression [7] reduces atherosclerosis by increasing cholesterol clearance rate via endothelial RCT. The endothelial-specific *Scarb1* knockout [9], on the other hand, reduces atherosclerosis by inhibiting the vv neovascularization that is essential for atherogenesis in the mouse model. Definitive proof of which interpretation is correct will require separating the two activities (i.e., LDL transcytosis and angiogenesis) in a suitable animal model.

The complete *cav1* knockout also inhibits atherosclerosis in *apoE*^−/−^ mice fed a high-fat diet [17] and endothelial LDL transcytosis in vitro [18]. The simple interpretation of these results has been that the loss of the Cav1 expression confers atheroprotection by eliminating SR-B1- and ALK1-mediated LDL transcytosis [92]. Nevertheless, again, Cav1 is also a regulator of angiogenesis, which is inhibited by the protein's deficiency [93], and the actual cause of reduced atherosclerosis in *cav1*^−/−^*apoE*^−/−^ double-knockout mice cannot be resolved. Cav1 deficiency might also reduce lipoproteins' passive transport into the arterial wall through existing vv capillaries - adding another confounding factor to the mix.

There are several other proteins, such as high mobility group box 1 (HMGB1), angiotensin II, and C-reactive protein (CRP), that enhance LDL transcytosis in vitro and atherosclerosis in either *apoE*^−/−^ or *ldlr*^−/−^ mice in vivo [71, 94, 95]. However, since all three also promote angiogenesis [96–98], the elucidation of their involvement in human atherogenesis will require a model in which LDL transcytosis and angiogenic activities can be untangled.

Another finding by Ghaffari et al. [99] is that in vitro, LDL transcytosis is negatively regulated by estrogen via activation of the G-protein-coupled estrogen receptor (GPER) that leads to inhibition of the SR-B1 endothelial expression. This finding seems to provide an elegant account of why premenopausal women are more protected against atherosclerosis and cardiovascular disease than age-matched men or postmenopausal women [100].

However, the reported experiments are challenging to interpret: endothelial primary culture cells derived from male and postmenopausal female donors and exposed to supraphysiological estrogen levels (0.5-1 nM) responded via the GPER by a concomitant dose-dependent reduction of the SR-B1 expression and LDL transcytosis rate; conversely, cells derived from premenopausal female donors, for which these estrogen levels were physiological, did not respond at all; although, the GPER signal-transduction pathway is their putative mechanism of estrogen atheroprotection. Hence, the observed reduction in the SR-B1 expression and LDL transcytosis in endothelial cells from premenopausal women are apparently not under estrogen control, and the molecular basis of estrogen atheroprotection cannot be reduced LDL transcytosis. The experimentally observed downregulation of the SR-B1 expression by supraphysiological estrogen in male- and postmenopausal female-derived cells [99] is probably nonphysiological and may only reflect the control that estrogen exerts over SR-B1 and the SR-B1-mediated influx of cholesterol into the steroidogenic ovaries of premenopausal women [101].

In agreement with our proposed TCF-dependent atherogenesis mechanism, we suggest that estrogen might specifically enhance cholesterol efflux from the arterial endothelium, similar to the enhancement that it exerts through its beta receptor (ER*β*) in VSMCs involving the cholesterol transporters ABCA1 and ABCG1, but not SR-B1 [102]. Alternatively, estrogen's beneficial effect could be a direct consequence of its powerful and lasting vasodilator activity [100], leading to lowered blood pressure and more efficient HDL-mediated endothelial RCT.

### 3.4. Important Implications of TCF-Driven Atherogenesis

#### 3.4.1. Media Thickness and Vasa Vasorum Extent Determine Atherosclerosis Susceptibility

The larger endothelial surface area-to-wall thickness ratio is probably why veins and small-diameter arteries, with a much thinner media and more laminar blood flow, remain atherosclerosis-free. On the other hand, arterialized vein grafts undergoing media thickening [103] and neovascularization and expansion of the vv to the intima [104] often fail due to atherosclerotic stenosis.

Arteries with little or no vv, such as the mammary and intramyocardial arteries, will also be less susceptible to atherosclerosis [44, 105], probably due to reduced influx of surplus cholesterol. If lipoproteins entered the arterial wall through the endothelium and exited through the lymphatic vv [52], vv-less arteries would be more susceptible to atherosclerosis per the current paradigm.

#### 3.4.2. Site Specificity of Atherosclerosis and HDL-Mediated Endothelial Efflux

Atherosclerosis is site-specific and more frequent in areas of low endothelial shear stress and turbulent blood flow [106], not because the turbulent flow “injures” the endothelium or limits oxygen transport to the vessel's wall [107], but because it exerts a greater force on the HDL particle and may disrupt cholesterol efflux by dislodging it from its receptor. Interestingly, caveolae number and Cav1 and SR-B1 expression are higher in atheroprone sections of the murine aorta where blood flow is turbulent [9, 18] in what looks like an effort to compensate for the harsher HDL-binding conditions.

#### 3.4.3. Hypertension and Atherosclerosis

Hypertension is a well-established cardiovascular risk factor and increases the risk of atherosclerosis [108]. Consistent with our proposed TCF mechanism, increased blood pressure and flow velocity can disturb endothelial RCT and accelerate atherogenesis in places where the flow becomes turbulent. Therefore, eNOS-deficient (eNOS^−/−^) mice exhibit accelerated atherogenesis [109], whereas certain blood-pressure-lowering drugs can slow it down and even cause regression of noncalcified plaques [110]. Conversely, RCT malfunction could decrease HDL concentrations, resulting in decreased HDL-mediated eNOS activation and increased blood pressure [111].

#### 3.4.4. The Hypoxia-Atherosclerosis Connection

Atherosclerosis and arterial wall hypoxia are associated [107]. The avascular media of the large arteries is always found on the verge of hypoxia - a problem that is exacerbated in sites of turbulent blood flow and low shear stress, where fluid mechanical effects limit oxygen transport from the lumen [107, 112]. Decreased oxygen tension was found in the inner 40% of the dog carotid artery wall at the atheroprone, low-shear-stress site of the carotid sinus [113]; the atherosclerotic wall of the rabbit aorta was found to be hypoxic, and hypoxia induced in the dog aorta by vv blockage was accompanied by medial necrosis [49, 114]. However, the mechanism underlying the hypoxia – atherosclerosis connection is still not adequately understood [107]. Large veins may be more hypoxic than arteries and yet remain atherosclerosis-free [107]. Here again, the TCF mechanism can offer a straightforward explanation: RCT malfunction in atheroprone regions could increase the endothelial membrane's cholesterol content, which may significantly reduce oxygen diffusion [22, 115]. This reduction (a phenomenon that surprisingly, seems to be unknown to the atherosclerosis research community) may cause hypoxia or even necrosis in media layers proximal to the lumen and far from the vv [40, 116]. Hypoxia, in turn, can induce neovascularization [117] and extension of the vv into the media and intima. Indeed, extensive neovascularization usually precedes plaque formation [41, 118]. The new blood vessels increase oxygen supply and the influx of LDL, possibly setting off a vicious cycle in which increased cholesterol deposition causes hypoxia, and hypoxia accelerates the arrival of cholesterol by inducing neovascularization. Hypoxia-induced necrosis could also create a similar runoff process by adding cholesterol released from dead cells to the TCF. In this case, the end result could be accelerated medial necrosis that may lead to an aneurysm - the other manifestation of vascular cholesterol homeostasis disorder [119].

## 4. Fighting Atherosclerosis by Targeting Its Root Cause Instead of Its Secondary Symptoms

The initial signs of atherosclerosis appear in medial VSMCs when their ability to maintain cholesterol homeostasis is probably compromised by unbalanced TCF and impaired RCT due to increased cholesterol influx through the vv or decreased endothelial efflux. The result is the early migration of VSMCs into areas with DIT, which later become sites of plaque formation. As cholesterol accumulates, initially at the media-intima boundary [69], these intimal VSMCs transdifferentiate into cholesterol-loaded foam cells. In fact, as mentioned above, most foam cells originate from VSMCs [34]. Therefore, the initiation of inflammation and the consequent infiltration of monocytes and their transformation to macrophages and then to foam cells seem to be only secondary symptoms triggered by lipids accumulation, oxidation, and enzymatic modification [84, 85].

The best argument for the pivotal role of macrophages in atherosclerosis, aside from their appearance on the scene, is the involvement of signaling molecules that affect macrophage proliferation in the development of the disease in mice. Glass and Witztum [120] listed several such cytokines and cytokine receptors, whose knockout almost completely inhibits atherosclerosis in *apoE*^−^/^−^ mice. However, it turns out that all of these molecules also similarly affect endothelial and smooth muscle cells proliferation and, in effect, the entire arterial wall (our survey of the literature). Hence, such knockout experiments cannot establish clear cause-effect relationships between macrophages and atherosclerosis, and efforts to inhibit macrophages' appearance or reduce their cholesterol load [121, 122] are likely to be ineffective, as they fail to target the primary cause. The same reasoning may also apply to (usually unsuccessful) attempts to fight atherosclerosis with antioxidants [123, 124].

Recent large-scale clinical trials have successfully demonstrated the efficacy of anti-inflammatory therapy in significantly reducing recurrent major adverse cardiovascular events in myocardial infarction patients by blocking specific immune modulators [125, 126]. However, given the enormous complexity of the immune system, the consequences of long-term use of anti-inflammatory drugs to prevent acute coronary syndrome are still unknown [127]. Although inflammation drives the progression of atherosclerosis and is responsible for its deadly outcome, the root cause remains the intimal accumulation of cholesterol [125]. Therefore, prevention of atherogenesis in its initial stages should remain the preferred treatment strategy. Such prevention could be achieved by either reducing the rate of cholesterol arrival through the vv or increasing its clearance rate through the apical endothelial membrane. While the rate of cholesterol entry is already being dealt with by the use of cholesterol-lowering drugs (i.e., statins), specifically enhancing the endothelial cholesterol efflux capacity through its various mechanisms is a potential atherosclerosis therapy that has yet to be explored. Possible courses of action could be the selective enhancement of lipid-poor, cholesterol efflux-promoting pre-beta HDL or HDL_3_ fractions [128, 129], or increasing the expression of cholesterol transporters in atheroprone regions of the endothelium by targeted transfection of their mRNAs [130].

## 5. Conclusion

This review presents an alternative TCF-centered interpretation of the newly available data regarding lipoproteins' endothelial transcytosis and atherogenesis. By connecting the many pieces of existing evidence into a simpler, coherent description of atherosclerosis etiology, the current paradigm of atherogenesis predicated on cholesterol entry from the lumen is changed. We assert that atherosclerosis is a consequence of the need to supply enough oxygen and nutrients to the avascular media of large arteries through the vv, which is inevitably accompanied by a rapid influx of excess cholesterol, whose main way back to the bloodstream is through the apical endothelial membrane. Plaque formation begins when the endothelium, with its limited surface area, can no longer cope with the flux of cholesterol arriving from the media.

We believe that this account of atherogenesis provides a decent alternative to existing hypotheses, including the new LDL transcytosis hypothesis. As such, it points to new, still unexplored potential therapy options - especially those addressing the endothelium's capacity for cholesterol efflux. It also opens the door for a much-needed debate in light of recent discoveries. Irrespective of the outcome, this debate should improve our understanding of atherogenesis and the ability to prevent and treat this deadly disease.

## Data Availability

Previously reported data were used to support this study added to Figure 1. The data are available at (doi:10.1161/01.ATV.10.3.477; doi:10.1016/0021-9150(77)90177-0 and doi:10.1111/j.1440-1827.1975.tb00149.x). These prior studies are cited at relevant places within the text as references [55, 57, and 60].
